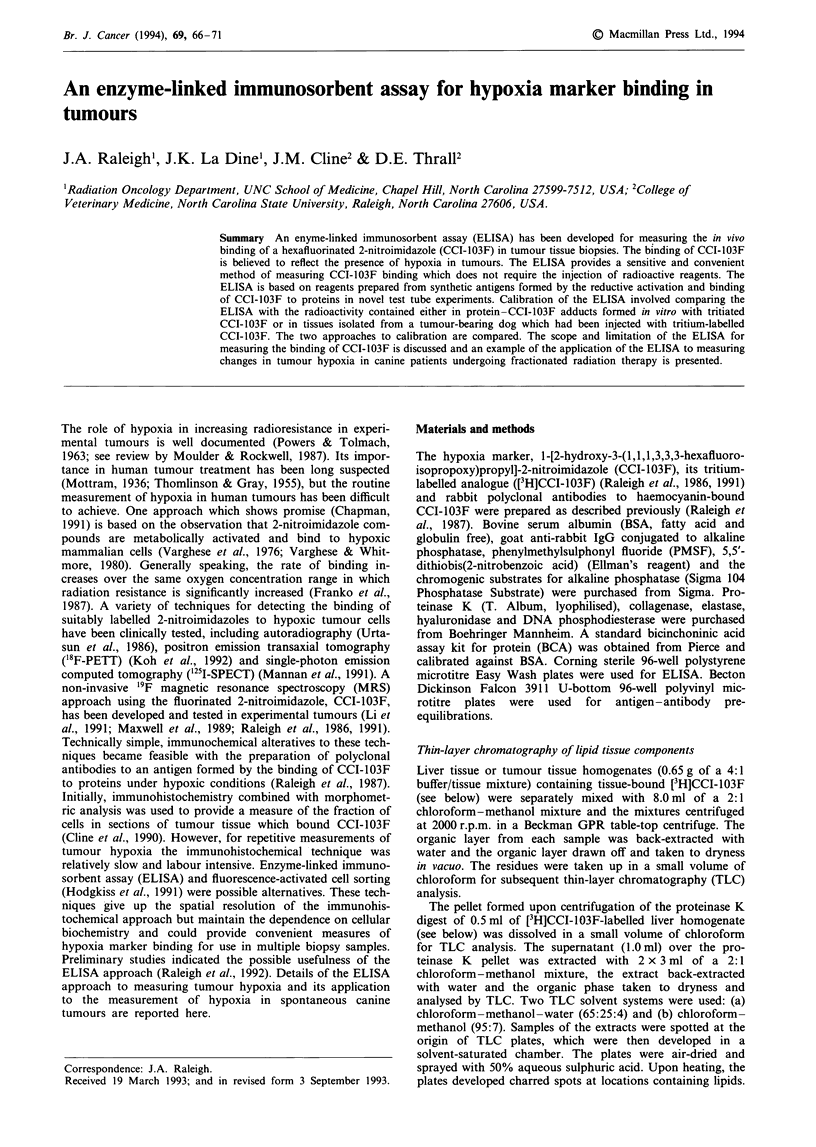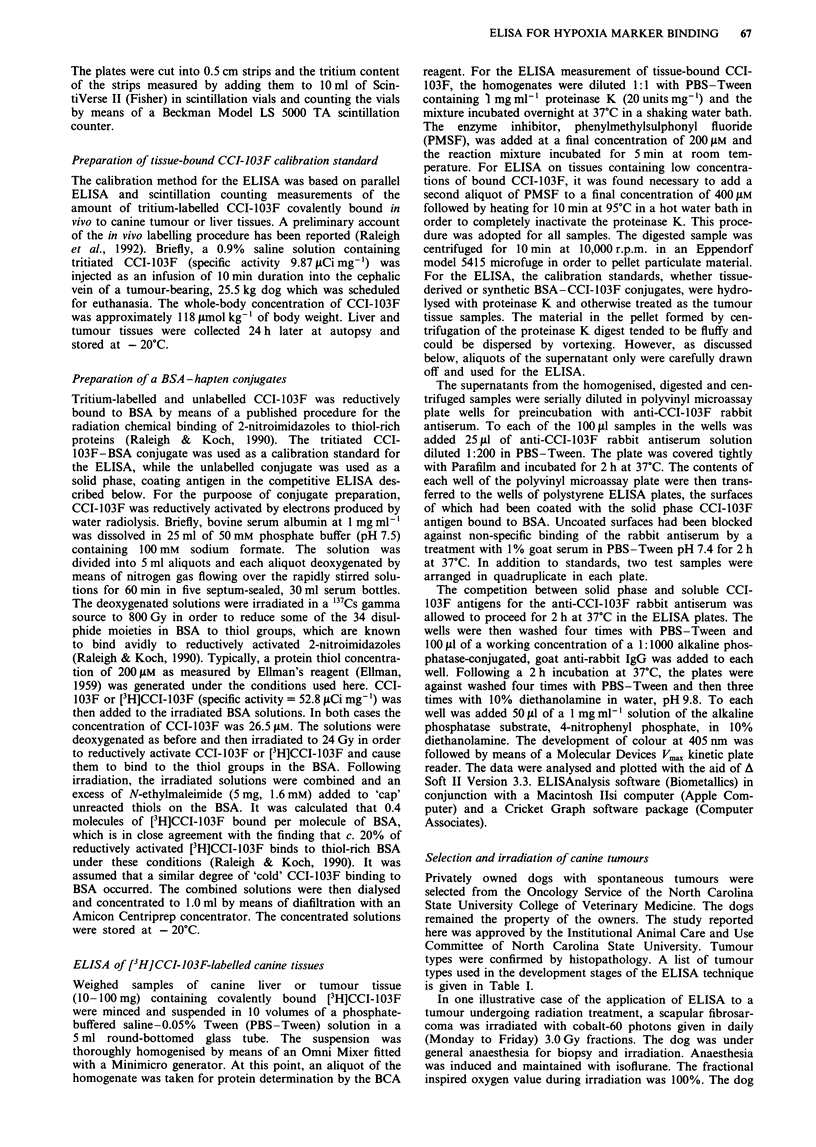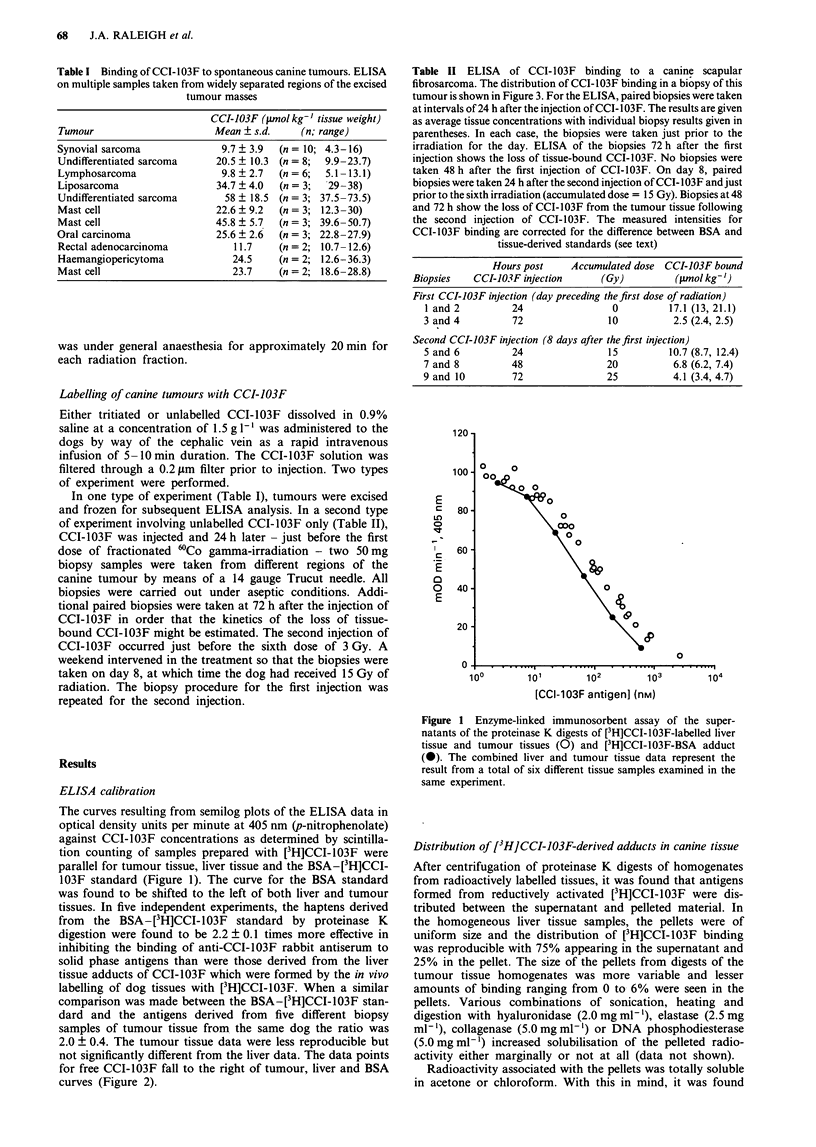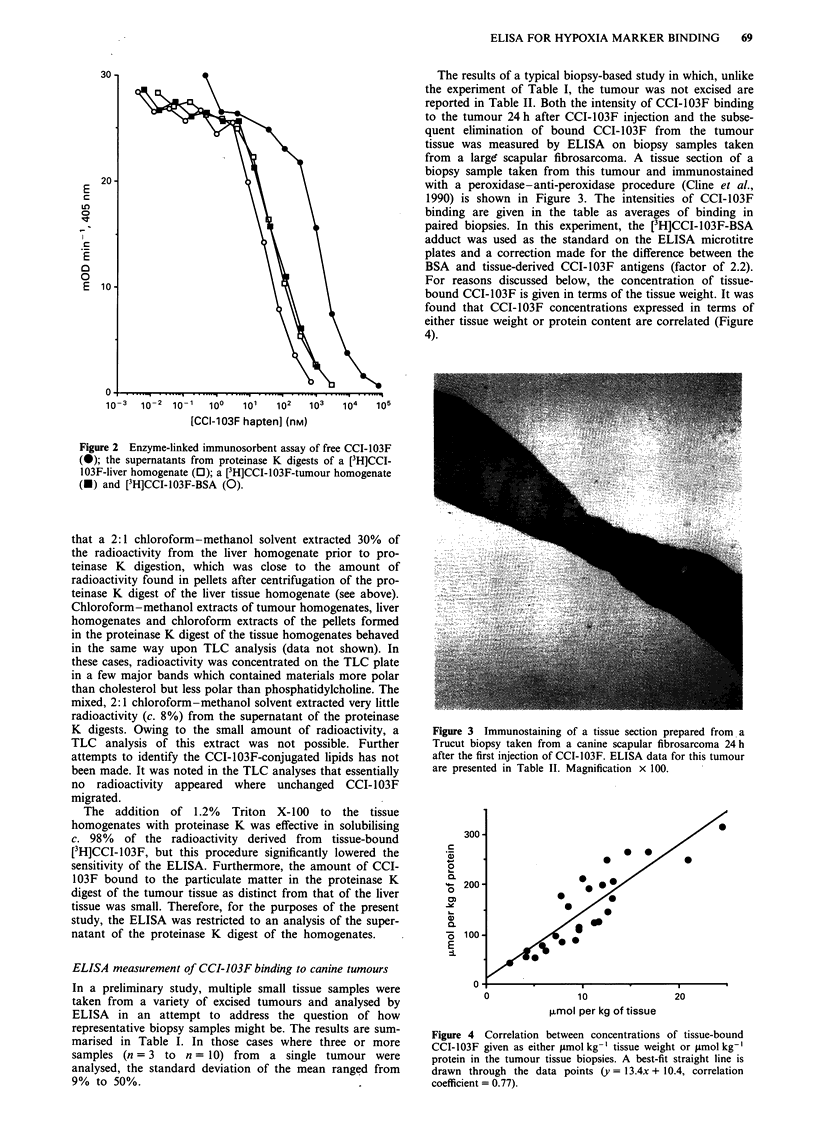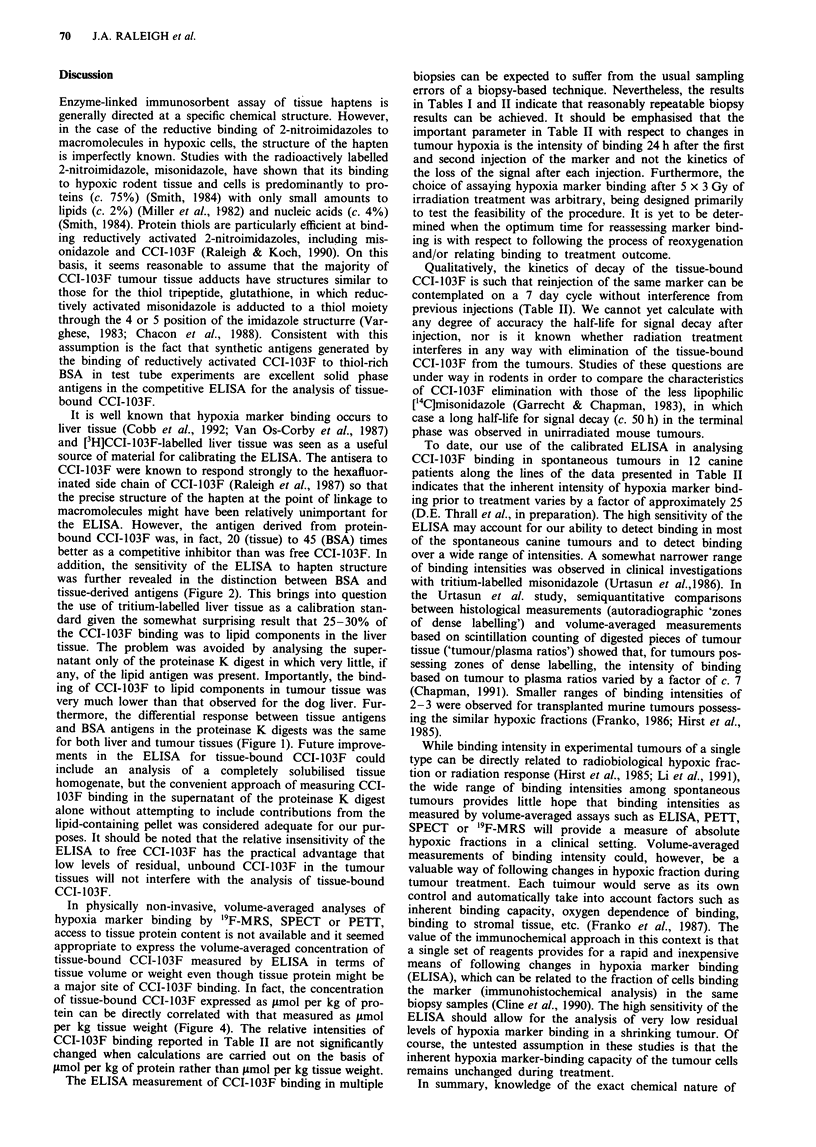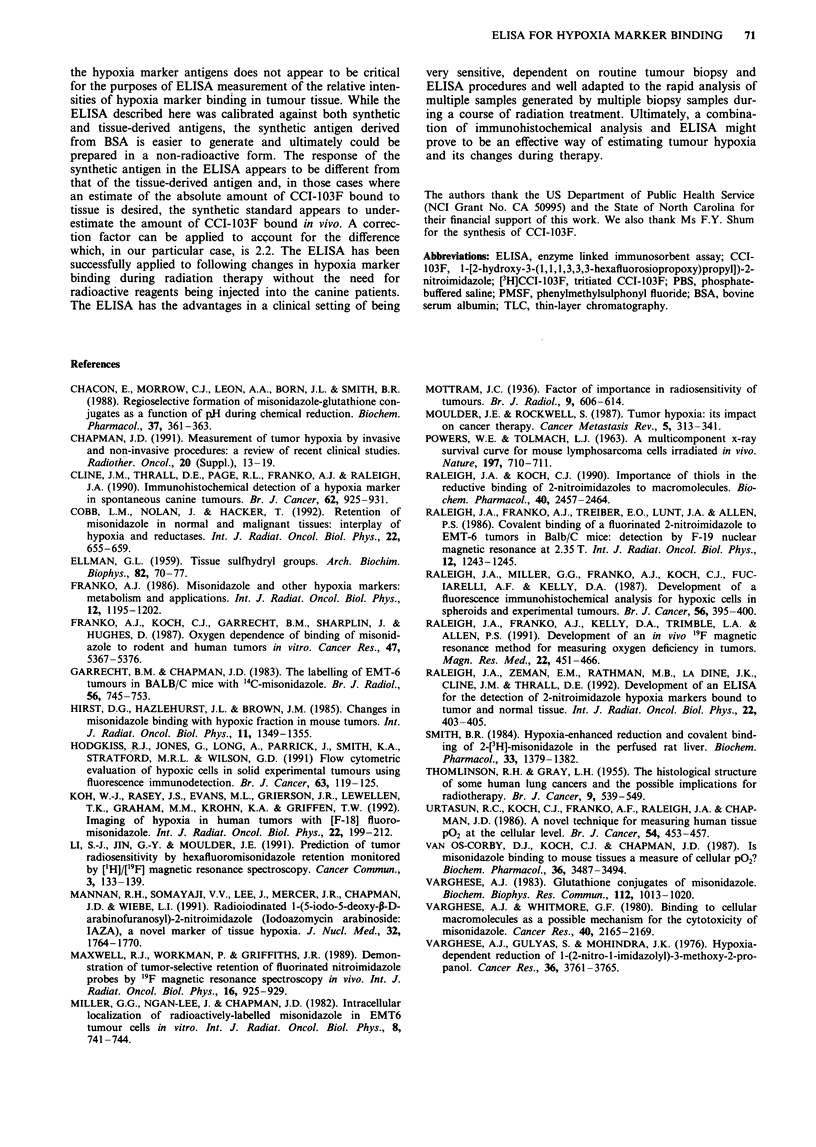# An enzyme-linked immunosorbent assay for hypoxia marker binding in tumours.

**DOI:** 10.1038/bjc.1994.10

**Published:** 1994-01

**Authors:** J. A. Raleigh, J. K. La Dine, J. M. Cline, D. E. Thrall

**Affiliations:** Radiation Oncology Department, UNC School of Medicine, Chapel Hill 27599-7512.

## Abstract

**Images:**


Br. J. Cancer (1994), 69, 66-71 © Macmillan Press Ltd., 1994

### An enzyme-linked immunosorbent assay for hypoxia marker binding in tumours

J.A. Raleigh[1], J.K. La Dine[1], J.M. Cline[2] & D.E. Thrall[2]

[1]*Radiation Oncology Department, UNC School of Medicine, Chapel Hill, North Carolina 27599-7512, USA;* [2]*College of Veterinary Medicine, North Carolina State University, Raleigh, North Carolina 27606, USA.*

**Summary** An enyme-linked immunosorbent assay (ELISA) has been developed for measuring the *in vivo* binding of a hexafluorinated 2-nitroimidazole (CCI-103F) in tumour tissue biopsies. The binding of CCI-103F is believed to reflect the presence of hypoxia in tumours. The ELISA provides a sensitive and convenient method of measuring CCI-103F binding which does not require the injection of radioactive reagents. The ELISA is based on reagents prepared from synthetic antigens formed by the reductive activation and binding of CCI-103F to proteins in novel test tube experiments. Calibration of the ELISA involved comparing the ELISA with the radioactivity contained either in protein-CCI-103F adducts formed *in vitro* with tritiated CCI-103F or in tissues isolated from a tumour-bearing dog which had been injected with tritium-labelled CCI-103F. The two approaches to calibration are compared. The scope and limitation of the ELISA for measuring the binding of CCI-103F is discussed and an example of the application of the ELISA to measuring changes in tumour hypoxia in canine patients undergoing fractionated radiation therapy is presented.

The role of hypoxia in increasing radioresistance in experimental tumours is well documented (Powers & Tolmach, 1963; see review by Moulder & Rockwell, 1987). Its importance in human tumour treatment has been long suspected (Mottram, 1936; Thomlinson & Gray, 1955), but the routine measurement of hypoxia in human tumours has been difficult to achieve. One approach which shows promise (Chapman, 1991) is based on the observation that 2-nitroimidazole compounds are metabolically activated and bind to hypoxic mammalian cells (Varghese *et al.*, 1976; Varghese & Whitmore, 1980). Generally speaking, the rate of binding increases over the same oxygen concentration range in which radiation resistance is significantly increased (Franko *et al.*, 1987). A variety of techniques for detecting the binding of suitably labelled 2-nitroimidazoles to hypoxic tumour cells have been clinically tested, including autoradiography (Urtasun *et al.*, 1986), positron emission transaxial tomography ($^{18}$F-PETT) (Koh *et al.*, 1992) and single-photon emission computed tomography ($^{125}$I-SPECT) (Mannan *et al.*, 1991). A non-invasive $^{19}$F magnetic resonance spectroscopy (MRS) approach using the fluorinated 2-nitroimidazole, CCI-103F, has been developed and tested in experimental tumours (Li *et al.*, 1991; Maxwell *et al.*, 1989; Raleigh *et al.*, 1986, 1991). Technically simple, immunochemical alteratives to these techniques became feasible with the preparation of polyclonal antibodies to an antigen formed by the binding of CCI-103F to proteins under hypoxic conditions (Raleigh *et al.*, 1987). Initially, immunohistochemistry combined with morphometric analysis was used to provide a measure of the fraction of cells in sections of tumour tissue which bound CCI-103F (Cline *et al.*, 1990). However, for repetitive measurements of tumour hypoxia the immunohistochemical technique was relatively slow and labour intensive. Enzyme-linked immunosorbent assay (ELISA) and fluorescence-activated cell sorting (Hodgkiss *et al.*, 1991) were possible alternatives. These techniques give up the spatial resolution of the immunohistochemical approach but maintain the dependence on cellular biochemistry and could provide convenient measures of hypoxia marker binding for use in multiple biopsy samples. Preliminary studies indicated the possible usefulness of the ELISA approach (Raleigh *et al.*, 1992). Details of the ELISA approach to measuring tumour hypoxia and its application to the measurement of hypoxia in spontaneous canine tumours are reported here.

Correspondence: J.A. Raleigh.
Received 19 March 1993; and in revised form 3 September 1993.

### Materials and methods

The hypoxia marker, 1-[2-hydroxy-3-(1,1,1,3,3,3-hexafluoroisopropoxy)propyl]-2-nitroimidazole (CCI-103F), its tritium-labelled analogue ([$^3$H]CCI-103F) (Raleigh *et al.*, 1986, 1991) and rabbit polyclonal antibodies to haemocyanin-bound CCI-103F were prepared as described previously (Raleigh *et al.*, 1987). Bovine serum albumin (BSA, fatty acid and globulin free), goat anti-rabbit IgG conjugated to alkaline phosphatase, phenylmethylsulphonyl fluoride (PMSF), 5,5'-dithiobis(2-nitrobenzoic acid) (Ellman's reagent) and the chromogenic substrates for alkaline phosphatase (Sigma 104 Phosphatase Substrate) were purchased from Sigma. Proteinase K (T. Album, lyophilised), collagenase, elastase, hyaluronidase and DNA phosphodiesterase were purchased from Boehringer Mannheim. A standard bicinchoninic acid assay kit for protein (BCA) was obtained from Pierce and calibrated against BSA. Corning sterile 96-well polystyrene microtitre Easy Wash plates were used for ELISA. Becton Dickinson Falcon 3911 U-bottom 96-well polyvinyl microtitre plates were used for antigen-antibody pre-equilibrations.

### *Thin-layer chromatography of lipid tissue components*

Liver tissue or tumour tissue homogenates (0.65 g of a 4:1 buffer/tissue mixture) containing tissue-bound [$^3$H]CCI-103F (see below) were separately mixed with 8.0 ml of a 2:1 chloroform-methanol mixture and the mixtures centrifuged at 2000 r.p.m. in a Beckman GPR table-top centrifuge. The organic layer from each sample was back-extracted with water and the organic layer drawn off and taken to dryness *in vacuo*. The residues were taken up in a small volume of chloroform for subsequent thin-layer chromatography (TLC) analysis.

The pellet formed upon centrifugation of the proteinase K digest of 0.5 ml of [$^3$H]CCI-103F-labelled liver homogenate (see below) was dissolved in a small volume of chloroform for TLC analysis. The supernatant (1.0 ml) over the proteinase K pellet was extracted with 2 × 3 ml of a 2:1 chloroform-methanol mixture, the extract back-extracted with water and the organic phase taken to dryness and analysed by TLC. Two TLC solvent systems were used: (a) chloroform-methanol-water (65:25:4) and (b) chloroform-methanol (95:7). Samples of the extracts were spotted at the origin of TLC plates, which were then developed in a solvent-saturated chamber. The plates were air-dried and sprayed with 50% aqueous sulphuric acid. Upon heating, the plates developed charred spots at locations containing lipids.

The plates were cut into 0.5 cm strips and the tritium content of the strips measured by adding them to 10 ml of ScintiVerse II (Fisher) in scintillation vials and counting the vials by means of a Beckman Model LS 5000 TA scintillation counter.

*Preparation of tissue-bound CCI-103F calibration standard*

The calibration method for the ELISA was based on parallel ELISA and scintillation counting measurements of the amount of tritium-labelled CCI-103F covalently bound *in vivo* to canine tumour or liver tissues. A preliminary account of the *in vivo* labelling procedure has been reported (Raleigh *et al.*, 1992). Briefly, a 0.9% saline solution containing tritiated CCI-103F (specific activity 9.87 $\mu$Ci mg$^{-1}$) was injected as an infusion of 10 min duration into the cephalic vein of a tumour-bearing, 25.5 kg dog which was scheduled for euthanasia. The whole-body concentration of CCI-103F was approximately 118 $\mu$mol kg$^{-1}$ of body weight. Liver and tumour tissues were collected 24 h later at autopsy and stored at $-20$°C.

*Preparation of a BSA–hapten conjugates*

Tritium-labelled and unlabelled CCI-103F was reductively bound to BSA by means of a published procedure for the radiation chemical binding of 2-nitroimidazoles to thiol-rich proteins (Raleigh & Koch, 1990). The tritiated CCI-103F–BSA conjugate was used as a calibration standard for the ELISA, while the unlabelled conjugate was used as a solid phase, coating antigen in the competitive ELISA described below. For the purpoose of conjugate preparation, CCI-103F was reductively activated by electrons produced by water radiolysis. Briefly, bovine serum albumin at 1 mg ml$^{-1}$ was dissolved in 25 ml of 50 mM phosphate buffer (pH 7.5) containing 100 mM sodium formate. The solution was divided into 5 ml aliquots and each aliquot deoxygenated by means of nitrogen gas flowing over the rapidly stirred solutions for 60 min in five septum-sealed, 30 ml serum bottles. The deoxygenated solutions were irradiated in a $^{137}$Cs gamma source to 800 Gy in order to reduce some of the 34 disulphide moieties in BSA to thiol groups, which are known to bind avidly to reductively activated 2-nitroimidazoles (Raleigh & Koch, 1990). Typically, a protein thiol concentration of 200 $\mu$M as measured by Ellman's reagent (Ellman, 1959) was generated under the conditions used here. CCI-103F or [$^3$H]CCI-103F (specific activity = 52.8 $\mu$Ci mg$^{-1}$) was then added to the irradiated BSA solutions. In both cases the concentration of CCI-103F was 26.5 $\mu$M. The solutions were deoxygenated as before and then irradiated to 24 Gy in order to reductively activate CCI-103F or [$^3$H]CCI-103F and cause them to bind to the thiol groups in the BSA. Following irradiation, the irradiated solutions were combined and an excess of *N*-ethylmaleimide (5 mg, 1.6 mM) added to 'cap' unreacted thiols on the BSA. It was calculated that 0.4 molecules of [$^3$H]CCI-103F bound per molecule of BSA, which is in close agreement with the finding that *c.* 20% of reductively activated [$^3$H]CCI-103F binds to thiol-rich BSA under these conditions (Raleigh & Koch, 1990). It was assumed that a similar degree of 'cold' CCI-103F binding to BSA occurred. The combined solutions were then dialysed and concentrated to 1.0 ml by means of diafiltration with an Amicon Centriprep concentrator. The concentrated solutions were stored at $-20$°C.

*ELISA of [$^3$H]CCI-103F-labelled canine tissues*

Weighed samples of canine liver or tumour tissue (10–100 mg) containing covalently bound [$^3$H]CCI-103F were minced and suspended in 10 volumes of a phosphate-buffered saline–0.05% Tween (PBS–Tween) solution in a 5 ml round-bottomed glass tube. The suspension was thoroughly homogenised by means of an Omni Mixer fitted with a Minimicro generator. At this point, an aliquot of the homogenate was taken for protein determination by the BCA reagent. For the ELISA measurement of tissue-bound CCI-103F, the homogenates were diluted 1:1 with PBS–Tween containing 1 mg ml$^{-1}$ proteinase K (20 units mg$^{-1}$) and the mixture incubated overnight at 37°C in a shaking water bath. The enzyme inhibitor, phenylmethylsulphonyl fluoride (PMSF), was added at a final concentration of 200 $\mu$M and the reaction mixture incubated for 5 min at room temperature. For ELISA on tissues containing low concentrations of bound CCI-103F, it was found necessary to add a second aliquot of PMSF to a final concentration of 400 $\mu$M followed by heating for 10 min at 95°C in a hot water bath in order to completely inactivate the proteinase K. This procedure was adopted for all samples. The digested sample was centrifuged for 10 min at 10,000 r.p.m. in an Eppendorf model 5415 microfuge in order to pellet particulate material. For the ELISA, the calibration standards, whether tissue-derived or synthetic BSA–CCI-103F conjugates, were hydrolysed with proteinase K and otherwise treated as the tumour tissue samples. The material in the pellet formed by centrifugation of the proteinase K digest tended to be fluffy and could be dispersed by vortexing. However, as discussed below, aliquots of the supernatant only were carefully drawn off and used for the ELISA.

The supernatants from the homogenised, digested and centrifuged samples were serially diluted in polyvinyl microassay plate wells for preincubation with anti-CCI-103F rabbit antiserum. To each of the 100 $\mu$l samples in the wells was added 25 $\mu$l of anti-CCI-103F rabbit antiserum solution diluted 1:200 in PBS–Tween. The plate was covered tightly with Parafilm and incubated for 2 h at 37°C. The contents of each well of the polyvinyl microassay plate were then transferred to the wells of polystyrene ELISA plates, the surfaces of which had been coated with the solid phase CCI-103F antigen bound to BSA. Uncoated surfaces had been blocked against non-specific binding of the rabbit antiserum by a treatment with 1% goat serum in PBS–Tween pH 7.4 for 2 h at 37°C. In addition to standards, two test samples were arranged in quadruplicate in each plate.

The competition between solid phase and soluble CCI-103F antigens for the anti-CCI-103F rabbit antiserum was allowed to proceed for 2 h at 37°C in the ELISA plates. The wells were then washed four times with PBS–Tween and 100 $\mu$l of a working concentration of a 1:1000 alkaline phosphatase-conjugated, goat anti-rabbit IgG was added to each well. Following a 2 h incubation at 37°C, the plates were against washed four times with PBS–Tween and then three times with 10% diethanolamine in water, pH 9.8. To each well was added 50 $\mu$l of a 1 mg ml$^{-1}$ solution of the alkaline phosphatase substrate, 4-nitrophenyl phosphate, in 10% diethanolamine. The development of colour at 405 nm was followed by means of a Molecular Devices $V_{max}$ kinetic plate reader. The data were analysed and plotted with the aid of Δ Soft II Version 3.3. ELISAnalysis software (Biometallics) in conjunction with a Macintosh IIsi computer (Apple Computer) and a Cricket Graph software package (Computer Associates).

*Selection and irradiation of canine tumours*

Privately owned dogs with spontaneous tumours were selected from the Oncology Service of the North Carolina State University College of Veterinary Medicine. The dogs remained the property of the owners. The study reported here was approved by the Institutional Animal Care and Use Committee of North Carolina State University. Tumour types were confirmed by histopathology. A list of tumour types used in the development stages of the ELISA technique is given in Table I.

In one illustrative case of the application of ELISA to a tumour undergoing radiation treatment, a scapular fibrosarcoma was irradiated with cobalt-60 photons given in daily (Monday to Friday) 3.0 Gy fractions. The dog was under general anaesthesia for biopsy and irradiation. Anaesthesia was induced and maintained with isoflurane. The fractional inspired oxygen value during irradiation was 100%. The dog

**Table I** Binding of CCI-103F to spontaneous canine tumours. ELISA on multiple samples taken from widely separated regions of the excised tumour masses

| Tumour | CCI-103F (μmol kg$^{-1}$ tissue weight) Mean ± s.d. | (n; range) |
|---|---|---|
| Synovial sarcoma | 9.7 ± 3.9 | (n = 10; 4.3–16) |
| Undifferentiated sarcoma | 20.5 ± 10.3 | (n = 8; 9.9–23.7) |
| Lymphosarcoma | 9.8 ± 2.7 | (n = 6; 5.1–13.1) |
| Liposarcoma | 34.7 ± 4.0 | (n = 3; 29–38) |
| Undifferentiated sarcoma | 58 ± 18.5 | (n = 3; 37.5–73.5) |
| Mast cell | 22.6 ± 9.2 | (n = 3; 12.3–30) |
| Mast cell | 45.8 ± 5.7 | (n = 3; 39.6–50.7) |
| Oral carcinoma | 25.6 ± 2.6 | (n = 3; 22.8–27.9) |
| Rectal adenocarcinoma | 11.7 | (n = 2; 10.7–12.6) |
| Haemangiopericytoma | 24.5 | (n = 2; 12.6–36.3) |
| Mast cell | 23.7 | (n = 2; 18.6–28.8) |

was under general anaesthesia for approximately 20 min for each radiation fraction.

*Labelling of canine tumours with CCI-103F*

Either tritiated or unlabelled CCI-103F dissolved in 0.9% saline at a concentration of 1.5 g l$^{-1}$ was administered to the dogs by way of the cephalic vein as a rapid intravenous infusion of 5–10 min duration. The CCI-103F solution was filtered through a 0.2 μm filter prior to injection. Two types of experiment were performed.

In one type of experiment (Table I), tumours were excised and frozen for subsequent ELISA analysis. In a second type of experiment involving unlabelled CCI-103F only (Table II), CCI-103F was injected and 24 h later – just before the first dose of fractionated $^{60}$Co gamma-irradiation – two 50 mg biopsy samples were taken from different regions of the canine tumour by means of a 14 gauge Trucut needle. All biopsies were carried out under aseptic conditions. Additional paired biopsies were taken at 72 h after the injection of CCI-103F in order that the kinetics of the loss of tissue-bound CCI-103F might be estimated. The second injection of CCI-103F occurred just before the sixth dose of 3 Gy. A weekend intervened in the treatment so that the biopsies were taken on day 8, at which time the dog had received 15 Gy of radiation. The biopsy procedure for the first injection was repeated for the second injection.

### Results

*ELISA calibration*

The curves resulting from semilog plots of the ELISA data in optical density units per minute at 405 nm (p-nitrophenolate) against CCI-103F concentrations as determined by scintillation counting of samples prepared with [$^3$H]CCI-103F were parallel for tumour tissue, liver tissue and the BSA-[$^3$H]CCI-103F standard (Figure 1). The curve for the BSA standard was found to be shifted to the left of both liver and tumour tissues. In five independent experiments, the haptens derived from the BSA-[$^3$H]CCI-103F standard by proteinase K digestion were found to be 2.2 ± 0.1 times more effective in inhibiting the binding of anti-CCI-103F rabbit antiserum to solid phase antigens than were those derived from the liver tissue adducts of CCI-103F which were formed by the *in vivo* labelling of dog tissues with [$^3$H]CCI-103F. When a similar comparison was made between the BSA-[$^3$H]CCI-103F standard and the antigens derived from five different biopsy samples of tumour tissue from the same dog the ratio was 2.0 ± 0.4. The tumour tissue data were less reproducible but not significantly different from the liver data. The data points for free CCI-103F fall to the right of tumour, liver and BSA curves (Figure 2).

**Table II** ELISA of CCI-103F binding to a canine scapular fibrosarcoma. The distribution of CCI-103F binding in a biopsy of this tumour is shown in Figure 3. For the ELISA, paired biopsies were taken at intervals of 24 h after the injection of CCI-103F. The results are given as average tissue concentrations with individual biopsy results given in parentheses. In each case, the biopsies were taken just prior to the irradiation for the day. ELISA of the biopsies 72 h after the first injection shows the loss of tissue-bound CCI-103F. No biopsies were taken 48 h after the first injection of CCI-103F. On day 8, paired biopsies were taken 24 h after the second injection of CCI-103F and just prior to the sixth irradiation (accumulated dose = 15 Gy). Biopsies at 48 and 72 h show the loss of CCI-103F from the tumour tissue following the second injection of CCI-103F. The measured intensities for CCI-103F binding are corrected for the difference between BSA and tissue-derived standards (see text)

| Biopsies | Hours post CCI-103F injection | Accumulated dose (Gy) | CCI-103F bound (μmol kg$^{-1}$) |
|---|---|---|---|
| *First CCI-103F injection (day preceding the first dose of radiation)* | | | |
| 1 and 2 | 24 | 0 | 17.1 (13, 21.1) |
| 3 and 4 | 72 | 10 | 2.5 (2.4, 2.5) |
| *Second CCI-103F injection (8 days after the first injection)* | | | |
| 5 and 6 | 24 | 15 | 10.7 (8.7, 12.4) |
| 7 and 8 | 48 | 20 | 6.8 (6.2, 7.4) |
| 9 and 10 | 72 | 25 | 4.1 (3.4, 4.7) |

**Figure 1** Enzyme-linked immunosorbent assay of the supernatants of the proteinase K digests of [$^3$H]CCI-103F-labelled liver tissue and tumour tissues (○) and [$^3$H]CCI-103F-BSA adduct (●). The combined liver and tumour tissue data represent the result from a total of six different tissue samples examined in the same experiment.

*Distribution of [$^3$H]CCI-103F-derived adducts in canine tissue*

After centrifugation of proteinase K digests of homogenates from radioactively labelled tissues, it was found that antigens formed from reductively activated [$^3$H]CCI-103F were distributed between the supernatant and pelleted material. In the homogeneous liver tissue samples, the pellets were of uniform size and the distribution of [$^3$H]CCI-103F binding was reproducible with 75% appearing in the supernatant and 25% in the pellet. The size of the pellets from digests of the tumour tissue homogenates was more variable and lesser amounts of binding ranging from 0 to 6% were seen in the pellets. Various combinations of sonication, heating and digestion with hyaluronidase (2.0 mg ml$^{-1}$), elastase (2.5 mg ml$^{-1}$), collagenase (5.0 mg ml$^{-1}$) or DNA phosphodiesterase (5.0 mg ml$^{-1}$) increased solubilisation of the pelleted radioactivity either marginally or not at all (data not shown).

Radioactivity associated with the pellets was totally soluble in acetone or chloroform. With this in mind, it was found

**Figure 2** Enzyme-linked immunosorbent assay of free CCI-103F (●); the supernatants from proteinase K digests of a [$^3$H]CCI-103F-liver homogenate (□); a [$^3$H]CCI-103F-tumour homogenate (■) and [$^3$H]CCI-103F-BSA (○).

that a 2:1 chloroform–methanol solvent extracted 30% of the radioactivity from the liver homogenate prior to proteinase K digestion, which was close to the amount of radioactivity found in pellets after centrifugation of the proteinase K digest of the liver tissue homogenate (see above). Chloroform–methanol extracts of tumour homogenates, liver homogenates and chloroform extracts of the pellets formed in the proteinase K digest of the tissue homogenates behaved in the same way upon TLC analysis (data not shown). In these cases, radioactivity was concentrated on the TLC plate in a few major bands which contained materials more polar than cholesterol but less polar than phosphatidylcholine. The mixed, 2:1 chloroform–methanol solvent extracted very little radioactivity (*c.* 8%) from the supernatant of the proteinase K digests. Owing to the small amount of radioactivity, a TLC analysis of this extract was not possible. Further attempts to identify the CCI-103F-conjugated lipids has not been made. It was noted in the TLC analyses that essentially no radioactivity appeared where unchanged CCI-103F migrated.

The addition of 1.2% Triton X-100 to the tissue homogenates with proteinase K was effective in solubilising *c.* 98% of the radioactivity derived from tissue-bound [$^3$H]CCI-103F, but this procedure significantly lowered the sensitivity of the ELISA. Furthermore, the amount of CCI-103F bound to the particulate matter in the proteinase K digest of the tumour tissue as distinct from that of the liver tissue was small. Therefore, for the purposes of the present study, the ELISA was restricted to an analysis of the supernatant of the proteinase K digest of the homogenates.

### *ELISA measurement of CCI-103F binding to canine tumours*

In a preliminary study, multiple small tissue samples were taken from a variety of excised tumours and analysed by ELISA in an attempt to address the question of how representative biopsy samples might be. The results are summarised in Table I. In those cases where three or more samples ($n = 3$ to $n = 10$) from a single tumour were analysed, the standard deviation of the mean ranged from 9% to 50%.

The results of a typical biopsy-based study in which, unlike the experiment of Table I, the tumour was not excised are reported in Table II. Both the intensity of CCI-103F binding to the tumour 24 h after CCI-103F injection and the subsequent elimination of bound CCI-103F from the tumour tissue was measured by ELISA on biopsy samples taken from a large scapular fibrosarcoma. A tissue section of a biopsy sample taken from this tumour and immunostained with a peroxidase–anti-peroxidase procedure (Cline *et al.*, 1990) is shown in Figure 3. The intensities of CCI-103F binding are given in the table as averages of binding in paired biopsies. In this experiment, the [$^3$H]CCI-103F-BSA adduct was used as the standard on the ELISA microtitre plates and a correction made for the difference between the BSA and tissue-derived CCI-103F antigens (factor of 2.2). For reasons discussed below, the concentration of tissue-bound CCI-103F is given in terms of the tissue weight. It was found that CCI-103F concentrations expressed in terms of either tissue weight or protein content are correlated (Figure 4).

**Figure 3** Immunostaining of a tissue section prepared from a Trucut biopsy taken from a canine scapular fibrosarcoma 24 h after the first injection of CCI-103F. ELISA data for this tumour are presented in Table II. Magnification × 100.

**Figure 4** Correlation between concentrations of tissue-bound CCI-103F given as either μmol kg$^{-1}$ tissue weight or μmol kg$^{-1}$ protein in the tumour tissue biopsies. A best-fit straight line is drawn through the data points ($y = 13.4x + 10.4$, correlation coefficient = 0.77).

### Discussion

Enzyme-linked immunosorbent assay of tissue haptens is generally directed at a specific chemical structure. However, in the case of the reductive binding of 2-nitroimidazoles to macromolecules in hypoxic cells, the structure of the hapten is imperfectly known. Studies with the radioactively labelled 2-nitroimidazole, misonidazole, have shown that its binding to hypoxic rodent tissue and cells is predominantly to proteins (*c.* 75%) (Smith, 1984) with only small amounts to lipids (*c.* 2%) (Miller *et al.*, 1982) and nucleic acids (*c.* 4%) (Smith, 1984). Protein thiols are particularly efficient at binding reductively activated 2-nitroimidazoles, including misonidazole and CCI-103F (Raleigh & Koch, 1990). On this basis, it seems reasonable to assume that the majority of CCI-103F tumour tissue adducts have structures similar to those for the thiol tripeptide, glutathione, in which reductively activated misonidazole is adducted to a thiol moiety through the 4 or 5 position of the imidazole structurre (Varghese, 1983; Chacon *et al.*, 1988). Consistent with this assumption is the fact that synthetic antigens generated by the binding of reductively activated CCI-103F to thiol-rich BSA in test tube experiments are excellent solid phase antigens in the competitive ELISA for the analysis of tissue-bound CCI-103F.

It is well known that hypoxia marker binding occurs to liver tissue (Cobb *et al.*, 1992; Van Os-Corby *et al.*, 1987) and [$^3$H]CCI-103F-labelled liver tissue was seen as a useful source of material for calibrating the ELISA. The antisera to CCI-103F were known to respond strongly to the hexafluorinated side chain of CCI-103F (Raleigh *et al.*, 1987) so that the precise structure of the hapten at the point of linkage to macromolecules might have been relatively unimportant for the ELISA. However, the antigen derived from protein-bound CCI-103F was, in fact, 20 (tissue) to 45 (BSA) times better as a competitive inhibitor than was free CCI-103F. In addition, the sensitivity of the ELISA to hapten structure was further revealed in the distinction between BSA and tissue-derived antigens (Figure 2). This brings into question the use of tritium-labelled liver tissue as a calibration standard given the somewhat surprising result that 25–30% of the CCI-103F binding was to lipid components in the liver tissue. The problem was avoided by analysing the supernatant only of the proteinase K digest in which very little, if any, of the lipid antigen was present. Importantly, the binding of CCI-103F to lipid components in tumour tissue was very much lower than that observed for the dog liver. Furthermore, the differential response between tissue antigens and BSA antigens in the proteinase K digests was the same for both liver and tumour tissues (Figure 1). Future improvements in the ELISA for tissue-bound CCI-103F could include an analysis of a completely solubilised tissue homogenate, but the convenient approach of measuring CCI-103F binding in the supernatant of the proteinase K digest alone without attempting to include contributions from the lipid-containing pellet was considered adequate for our purposes. It should be noted that the relative insensitivity of the ELISA to free CCI-103F has the practical advantage that low levels of residual, unbound CCI-103F in the tumour tissues will not interfere with the analysis of tissue-bound CCI-103F.

In physically non-invasive, volume-averaged analyses of hypoxia marker binding by $^{19}$F-MRS, SPECT or PETT, access to tissue protein content is not available and it seemed appropriate to express the volume-averaged concentration of tissue-bound CCI-103F measured by ELISA in terms of tissue volume or weight even though tissue protein might be a major site of CCI-103F binding. In fact, the concentration of tissue-bound CCI-103F expressed as μmol per kg of protein can be directly correlated with that measured as μmol per kg tissue weight (Figure 4). The relative intensities of CCI-103F binding reported in Table II are not significantly changed when calculations are carried out on the basis of μmol per kg of protein rather than μmol per kg tissue weight.

The ELISA measurement of CCI-103F binding in multiple biopsies can be expected to suffer from the usual sampling errors of a biopsy-based technique. Nevertheless, the results in Tables I and II indicate that reasonably repeatable biopsy results can be achieved. It should be emphasised that the important parameter in Table II with respect to changes in tumour hypoxia is the intensity of binding 24 h after the first and second injection of the marker and not the kinetics of the loss of the signal after each injection. Furthermore, the choice of assaying hypoxia marker binding after 5 × 3 Gy of irradiation treatment was arbitrary, being designed primarily to test the feasibility of the procedure. It is yet to be determined when the optimum time for reassessing marker binding is with respect to following the process of reoxygenation and/or relating binding to treatment outcome.

Qualitatively, the kinetics of decay of the tissue-bound CCI-103F is such that reinjection of the same marker can be contemplated on a 7 day cycle without interference from previous injections (Table II). We cannot yet calculate with any degree of accuracy the half-life for signal decay after injection, nor is it known whether radiation treatment interferes in any way with elimination of the tissue-bound CCI-103F from the tumours. Studies of these questions are under way in rodents in order to compare the characteristics of CCI-103F elimination with those of the less lipophilic [$^{14}$C]misonidazole (Garrecht & Chapman, 1983), in which case a long half-life for signal decay (*c.* 50 h) in the terminal phase was observed in unirradiated mouse tumours.

To date, our use of the calibrated ELISA in analysing CCI-103F binding in spontaneous tumours in 12 canine patients along the lines of the data presented in Table II indicates that the inherent intensity of hypoxia marker binding prior to treatment varies by a factor of approximately 25 (D.E. Thrall *et al.*, in preparation). The high sensitivity of the ELISA may account for our ability to detect binding in most of the spontaneous canine tumours and to detect binding over a wide range of intensities. A somewhat narrower range of binding intensities was observed in clinical investigations with tritium-labelled misonidazole (Urtasun *et al.*,1986). In the Urtasun *et al.* study, semiquantitative comparisons between histological measurements (autoradiographic 'zones of dense labelling') and volume-averaged measurements based on scintillation counting of digested pieces of tumour tissue ('tumour/plasma ratios') showed that, for tumours possessing zones of dense labelling, the intensity of binding based on tumour to plasma ratios varied by a factor of *c.* 7 (Chapman, 1991). Smaller ranges of binding intensities of 2–3 were observed for transplanted murine tumours possessing the similar hypoxic fractions (Franko, 1986; Hirst *et al.*, 1985).

While binding intensity in experimental tumours of a single type can be directly related to radiobiological hypoxic fraction or radiation response (Hirst *et al.*, 1985; Li *et al.*, 1991), the wide range of binding intensities among spontaneous tumours provides little hope that binding intensities as measured by volume-averaged assays such as ELISA, PETT, SPECT or $^{19}$F-MRS will provide a measure of absolute hypoxic fractions in a clinical setting. Volume-averaged measurements of binding intensity could, however, be a valuable way of following changes in hypoxic fraction during tumour treatment. Each tuimour would serve as its own control and automatically take into account factors such as inherent binding capacity, oxygen dependence of binding, binding to stromal tissue, etc. (Franko *et al.*, 1987). The value of the immunochemical approach in this context is that a single set of reagents provides for a rapid and inexpensive means of following changes in hypoxia marker binding (ELISA), which can be related to the fraction of cells binding the marker (immunohistochemical analysis) in the same biopsy samples (Cline *et al.*, 1990). The high sensitivity of the ELISA should allow for the analysis of very low residual levels of hypoxia marker binding in a shrinking tumour. Of course, the untested assumption in these studies is that the inherent hypoxia marker-binding capacity of the tumour cells remains unchanged during treatment.

In summary, knowledge of the exact chemical nature of the hypoxia marker antigens does not appear to be critical for the purposes of ELISA measurement of the relative intensities of hypoxia marker binding in tumour tissue. While the ELISA described here was calibrated against both synthetic and tissue-derived antigens, the synthetic antigen derived from BSA is easier to generate and ultimately could be prepared in a non-radioactive form. The response of the synthetic antigen in the ELISA appears to be different from that of the tissue-derived antigen and, in those cases where an estimate of the absolute amount of CCI-103F bound to tissue is desired, the synthetic standard appears to underestimate the amount of CCI-103F bound *in vivo*. A correction factor can be applied to account for the difference which, in our particular case, is 2.2. The ELISA has been successfully applied to following changes in hypoxia marker binding during radiation therapy without the need for radioactive reagents being injected into the canine patients. The ELISA has the advantages in a clinical setting of being very sensitive, dependent on routine tumour biopsy and ELISA procedures and well adapted to the rapid analysis of multiple samples generated by multiple biopsy samples during a course of radiation treatment. Ultimately, a combination of immunohistochemical analysis and ELISA might prove to be an effective way of estimating tumour hypoxia and its changes during therapy.

The authors thank the US Department of Public Health Service (NCI Grant No. CA 50995) and the State of North Carolina for their financial support of this work. We also thank Ms F.Y. Shum for the synthesis of CCI-103F.

**Abbreviations:** ELISA, enzyme linked immunosorbent assay; CCI-103F, 1-[2-hydroxy-3-(1,1,1,3,3,3-hexafluorosiopropoxy)propyl])-2-nitroimidazole; [$^3$H]CCI-103F, tritiated CCI-103F; PBS, phosphate-buffered saline; PMSF, phenylmethylsulphonyl fluoride; BSA, bovine serum albumin; TLC, thin-layer chromatography.

### References

CHACON, E., MORROW, C.J., LEON, A.A., BORN, J.L. & SMITH, B.R. (1988). Regioselective formation of misonidazole-glutathione conjugates as a function of pH during chemical reduction. *Biochem. Pharmacol.*, **37**, 361–363.

CHAPMAN, J.D. (1991). Measurement of tumor hypoxia by invasive and non-invasive procedures: a review of recent clinical studies. *Radiother. Oncol.*, **20** (Suppl.), 13–19.

CLINE, J.M., THRALL, D.E., PAGE, R.L., FRANKO, A.J. & RALEIGH, J.A. (1990). Immunohistochemical detection of a hypoxia marker in spontaneous canine tumours. *Br. J. Cancer*, **62**, 925–931.

COBB, L.M., NOLAN, J. & HACKER, T. (1992). Retention of misonidazole in normal and malignant tissues: interplay of hypoxia and reductases. *Int. J. Radiat. Oncol. Biol. Phys.*, **22**, 655–659.

ELLMAN, G.L. (1959). Tissue sulfhydryl groups. *Arch. Biochim. Biophys.*, **82**, 70–77.

FRANKO, A.J. (1986). Misonidazole and other hypoxia markers: metabolism and applications. *Int. J. Radiat. Oncol. Biol. Phys.*, **12**, 1195–1202.

FRANKO, A.J., KOCH, C.J., GARRECHT, B.M., SHARPLIN, J. & HUGHES, D. (1987). Oxygen dependence of binding of misonidazole to rodent and human tumors *in vitro*. *Cancer Res.*, **47**, 5367–5376.

GARRECHT, B.M. & CHAPMAN, J.D. (1983). The labelling of EMT-6 tumours in BALB/C mice with $^{14}$C-misonidazole. *Br. J. Radiol.*, **56**, 745–753.

HIRST, D.G., HAZLEHURST, J.L. & BROWN, J.M. (1985). Changes in misonidazole binding with hypoxic fraction in mouse tumors. *Int. J. Radiat. Oncol. Biol. Phys.*, **11**, 1349–1355.

HODGKISS, R.J., JONES, G., LONG, A., PARRICK, J., SMITH, K.A., STRATFORD, M.R.L. & WILSON, G.D. (1991) Flow cytometric evaluation of hypoxic cells in solid experimental tumours using fluorescence immunodetection. *Br. J. Cancer*, **63**, 119–125.

KOH, W.-J., RASEY, J.S., EVANS, M.L., GRIERSON, J.R., LEWELLEN, T.K., GRAHAM, M.M., KROHN, K.A. & GRIFFEN, T.W. (1992). Imaging of hypoxia in human tumors with [F-18] fluoromisonidazole. *Int. J. Radiat. Oncol. Biol. Phys.*, **22**, 199–212.

LI, S.-J., JIN, G.-Y. & MOULDER, J.E. (1991). Prediction of tumor radiosensitivity by hexafluoromisonidazole retention monitored by [$^1$H]/[$^{19}$F] magnetic resonance spectroscopy. *Cancer Commun.*, **3**, 133–139.

MANNAN, R.H., SOMAYAJI, V.V., LEE, J., MERCER, J.R., CHAPMAN, J.D. & WIEBE, L.I. (1991). Radioiodinated 1-(5-iodo-5-deoxy-β-D-arabinofuranosyl)-2-nitroimidazole (Iodoazomycin arabinoside: IAZA), a novel marker of tissue hypoxia. *J. Nucl. Med.*, **32**, 1764–1770.

MAXWELL, R.J., WORKMAN, P. & GRIFFITHS, J.R. (1989). Demonstration of tumor-selective retention of fluorinated nitroimidazole probes by $^{19}$F magnetic resonance spectroscopy *in vivo*. *Int. J. Radiat. Oncol. Biol. Phys.*, **16**, 925–929.

MILLER, G.G., NGAN-LEE, J. & CHAPMAN, J.D. (1982). Intracellular localization of radioactively-labelled misonidazole in EMT6 tumour cells *in vitro*. *Int. J. Radiat. Oncol. Biol. Phys.*, **8**, 741–744.

MOTTRAM, J.C. (1936). Factor of importance in radiosensitivity of tumours. *Br. J. Radiol.*, **9**, 606–614.

MOULDER, J.E. & ROCKWELL, S. (1987). Tumor hypoxia: its impact on cancer therapy. *Cancer Metastasis Rev.*, **5**, 313–341.

POWERS, W.E. & TOLMACH, L.J. (1963). A multicomponent x-ray survival curve for mouse lymphosarcoma cells irradiated *in vivo*. *Nature*, **197**, 710–711.

RALEIGH, J.A. & KOCH, C.J. (1990). Importance of thiols in the reductive binding of 2-nitroimidazoles to macromolecules. *Biochem. Pharmacol.*, **40**, 2457–2464.

RALEIGH, J.A., FRANKO, A.J., TREIBER, E.O., LUNT, J.A. & ALLEN, P.S. (1986). Covalent binding of a fluorinated 2-nitroimidazole to EMT-6 tumors in Balb/C mice: detection by F-19 nuclear magnetic resonance at 2.35 T. *Int. J. Radiat. Oncol. Biol. Phys.*, **12**, 1243–1245.

RALEIGH, J.A., MILLER, G.G., FRANKO, A.J., KOCH, C.J., FUCIARELLI, A.F. & KELLY, D.A. (1987). Development of a fluorescence immunohistochemical analysis for hypoxic cells in spheroids and experimental tumours. *Br. J. Cancer*, **56**, 395–400.

RALEIGH, J.A., FRANKO, A.J., KELLY, D.A., TRIMBLE, L.A. & ALLEN, P.S. (1991). Development of an *in vivo* $^{19}$F magnetic resonance method for measuring oxygen deficiency in tumors. *Magn. Res. Med.*, **22**, 451–466.

RALEIGH, J.A., ZEMAN, E.M., RATHMAN, M.B., LA DINE, J.K., CLINE, J.M. & THRALL, D.E. (1992). Development of an ELISA for the detection of 2-nitroimidazole hypoxia markers bound to tumor and normal tissue. *Int. J. Radiat. Oncol. Biol. Phys.*, **22**, 403–405.

SMITH, B.R. (1984). Hypoxia-enhanced reduction and covalent binding of 2-[$^3$H]-misonidazole in the perfused rat liver. *Biochem. Pharmacol.*, **33**, 1379–1382.

THOMLINSON, R.H. & GRAY, L.H. (1955). The histological structure of some human lung cancers and the possible implications for radiotherapy. *Br. J. Cancer*, **9**, 539–549.

URTASUN, R.C., KOCH, C.J., FRANKO, A.F., RALEIGH, J.A. & CHAPMAN, J.D. (1986). A novel technique for measuring human tissue $pO_2$ at the cellular level. *Br. J. Cancer*, **54**, 453–457.

VAN OS-CORBY, D.J., KOCH, C.J. & CHAPMAN, J.D. (1987). Is misonidazole binding to mouse tissues a measure of cellular $pO_2$? *Biochem. Pharmacol.*, **36**, 3487–3494.

VARGHESE, A.J. (1983). Glutathione conjugates of misonidazole. *Biochem. Biophys. Res. Commun.*, **112**, 1013–1020.

VARGHESE, A.J. & WHITMORE, G.F. (1980). Binding to cellular macromolecules as a possible mechanism for the cytotoxicity of misonidazole. *Cancer Res.*, **40**, 2165–2169.

VARGHESE, A.J., GULYAS, S. & MOHINDRA, J.K. (1976). Hypoxia-dependent reduction of 1-(2-nitro-1-imidazolyl)-3-methoxy-2-propanol. *Cancer Res.*, **36**, 3761–3765.